# Developing innovative targeted therapies for China

**DOI:** 10.1186/1479-5876-10-S2-A3

**Published:** 2012-10-17

**Authors:** Bahija Jallal

**Affiliations:** 1 Research and Development, MedImmune, LLC, Gaithersburg, MD, 20878, USA

## Background

MedImmune is the biologics business unit of AstraZeneca with a mission of helping patients with significant unmet medical need by developing innovative medicines. MedImmune has a rich pipeline across five therapeutic areas including oncology, respiratory, inflammatory and autoimmunity, infectious diseases, neurosciences, cardiovascular and gastrointestinal diseases. To realize our vision of personalized healthcare—treating the right patient with the right drug at the right dose, we are applying cutting edge tools such as next-gen sequencing, circulating tumor cells and advanced PK/PD modelling across the pipeline.

## Materials and methods

Fundamental to our goal of developing personalized healthcare is gaining deep understanding of diseases across different populations and specifically in Asians. To this end, we have formed partnerships with Chinese academic institutions, government agencies and industry partners, and have a history of working with scientists/physicians in China to develop medications that target the Asian/Chinese patient population. For example, the AstraZeneca product IRESSA (gefitinib), is already frontline therapy in China for the treatment of patients with locally advanced or metastatic non-small cell lung cancer with an activating mutation in the EGFR gene.

## Results

Most recently we set up an exciting collaboration with the Shanghai Chest and Renji Hospitals to develop a richly annotated database of lung cancer and hepatocellular cancer patient samples to aid in identifying novel drug targets and developing therapeutic strategies to aid patients. This presentation will provide examples in our pipeline that give us reasons to believe in such approaches, e.g. a type I interferon gene signature for an anti-IFN-alpha or anti-IFNAR monoclonal antibody in patients with systemic lupus erythematosus.

## Conclusions

MedImmune is committed to helping Chinese patients with significant unmet medical need and we believe collaborations with leading Chinese PIs and innovation are key to our mission.

